# Subsurface geophysical profiling in Awash Melkesa: Insights into lithology and resource potential

**DOI:** 10.1371/journal.pone.0333941

**Published:** 2025-10-06

**Authors:** Eyayu Ayalew Dessalew, Tilahun Mamo, Mohammed Yesuf Ali

**Affiliations:** 1 Department of Geology, College of Natural Science, Wollo University, Dessie, Ethiopia; 2 School of Earth science, College of Science, Addis Ababa University, Addis Ababa, Ethiopia; Wadia Institute of Himalayan Geology, INDIA

## Abstract

Integrated geophysical investigations were conducted around Awash Melkesa, located 106 km southeast of Addis Ababa, utilizing electrical resistivity, magnetic, radiometric, and seismic refraction methods. The geo-electric profile revealed five layers, with a notable low resistivity zone (113 Ohm-m) beneath VES 2, indicating a highly fractured and weathered ignimbrite, which suggests good potential for aquifers. The residual magnetic anomaly map varied from −312–296 nT, pointing to alluvial and pyroclastic sediments, while the tilt derivative magnetic map identified several faults oriented in N-S and NE-SW directions, consistent with the Wonji fault belt. Additionally, high concentrations of uranium and thorium were found in areas with felsic and intermediate extrusive igneous rocks, whereas lower concentrations were associated with weathered felsic rocks. The seismic velocity model identified three layers: the top layer, 3 m thick, showed P-wave velocities of 0.3–0.7 km/s; the second layer, at depths of 3–9 m, had velocities of 0.75–1.2 km/s, likely representing unconsolidated pyroclastic ignimbrite; and the third layer, between 9–18 m, exhibited a P-wave velocity of 2 km/s, indicating somewhat weathered and fractured ignimbrite. These findings provide valuable insights into the subsurface geophysical characteristics and potential resources in the Awash Melkesa area.

## 1. Introduction

Utilising electrical, magnetic, radiometric, and seismic refraction techniques, integrated geophysical methods have been used to measure subsurface anomalies in the Awash Melkesa area. Subsurface potentials and currents are evaluated using electrical resistivity techniques. Specifically, two basic techniques such as vertical electrical sounding (VES) and profiling are utilised to determine resistivity variations. VES detects vertical resistivity variations, while profiling provides horizontal resistivity profiles through methods such as Constant Separation Traversing (CST) and Electrical Resistivity Tomography (ERT). Despite the use of Dipole-Dipole techniques in the field investigation, no information was gathered to identify geophysical anomalies.

Magnetic surveys it makes of the differences in rock magnetisation, which are impacted by trace elements that have a big impact on magnetic properties. Finding anomalous magnetisation is its primary objective since it is essential for solving geophysical, hydrogeological, and engineering problems [1]. The overall field, including the anomalous field and other magnetic influences, is reflected in the observed data during surveys. Magnetic anomalies between the Dera and Sire regions were identified using this method.

Additionally, the three-line profile was used to map magnetic anomalies and combine the findings with other geophysical techniques. A radiometric survey was also carried out with an emphasis on gamma rays released by rocks and soils. The natural decay of uranium, thorium, and potassium produces the detectable gamma radiation that is essential for figuring out the composition of the subsoil [2].

One of the most useful surface geophysical techniques for determining the properties of underlying lithology is the seismic refraction survey [3]. The determination of bed rock physical properties, oil and gas exploration, groundwater exploration, lithology, subsurface structure, folds, and faults all use the seismic refraction approach. In order to identify subsurface structures, conduct groundwater research, and determine lithological features, seismic refraction surveys are a crucial method [4]. By passing through the crust and refracting or reflecting at geological boundaries, controlled seismic waves make it possible to map subsurface interfaces using arrival timings that are recorded by devices on the surface.

### 1.2. Location and accessibility

The study area, Awash Melkassa is located near to Adama city and is accessible on an asphalt road from Addis Ababa – Adama – Assela main road. It is located on the main road of Sodare, one of the famous recreational centers and easily accessible area of the country. It is bounded between UTM coordinate 535100–535900 Easting and 929900–930600 Northing which is part of the Main Ethiopian Rift (MER). More specifically, it is located in East shoa Zone of Oromia Regional state at about 106 km, SE of Addis Ababa and at around 14 km, SE of Adama city.

## 2. Materials and methods

### 2.1. Electrical methods

Electrical surveys are used to estimate actual resistivity by using surface data to ascertain the subsurface resistivity distribution. Geological elements like water saturation, porosity, and mineral and fluid content all affect ground resistivity [4,5]. Hydrogeological, mining, geotechnical [6–8], and more recently, environmental studies [2] have all made extensive use of electrical resistivity surveys.

Sounding surveys and profiling surveys are the two main measurement techniques; they vary in their intended emphasis and design. The main way that rock conduction happens in vertical electrical sounding (VES) is through pore fluids functioning as electrolytes. The presence of metallic minerals, clay content, water resistivity, and water content (porosity) all affect the resistivity of rocks [4,5].

Using the PASi 16GL model earth resistivity meter, three vertical electrical sounding (VES) surveys were carried out along this profile in order to comprehend the subsurface conditions of the research area. With sounding sites spaced an average of 100 meters apart, the survey profile was orientated from north to south. To investigate anisotropic responses and prevent data ambiguity, repeated readings were made at AB/2 distances of 20, 30, 150 m, and 220 m, respectively. GPS was used to record each sounding point’s position. To determine the first model parameters of potential layers from field VES data, IP2WIN software processes several key parameters. These include the calculation of apparent resistivity from voltage and current measurements, as well as estimating layer thickness and resistivity values for different geological units. The software employs curve fitting techniques to match theoretical models with observed data, adjusting parameters to improve accuracy. Additionally, local geological context is considered to ensure the results are geologically plausible. Error analysis is conducted to assess the reliability of the model, ultimately generating a resistivity model that accurately reflects the subsurface layers.

### 2.2. Magnetic method

Magnetic method is a passive geophysical method (use the natural field of the earth) involves the measurement of the earth’s magnetic field intensity to investigate subsurface geology on the basis of anomalies in the earth’s magnetic field resulting from the magnetic properties of the underlying rocks [9]. The magnetic method of prospecting has a great deal in common with the gravitational method. It has a broad range of applications, from small scale engineering or archaeological surveys to detect buried metallic objects, to large scale surveys carried out to investigate regional geological structures [9]. The magnetic survey was conducted using proton precision magnetometer and about 134 primary total intensity magnetic field measurements were collected. It is an automatic recording instrument with a resolution and absolute accuracy of 0.01 and 0.02 nT respectively over its full range temperature. It has three basic separate elements: reading and recording instrument, sensor and GPS. The primary data were collected with an average station interval of 10m and approximately NNW-SSE profiles. We have also conducted magnetic survey using the proton precision magnetometer along one profile line (20 km) with 2 km spacing from town Dera to Sire town along the main road.

### 2.3. Radioactive methods

One of the fastest and most affordable methods for geochemical mapping that takes into account the distribution of the radioactive elements such as potassium, uranium, and thorium, is the radiometric approach. The technique is now primarily used for geological mapping and the exploration of various forms of economic minerals; geochemical and environmental monitoring enable the analysis of regional features across vast areas and are applicable to many scientific domains [10,11].

Only potassium and the uranium and thorium decay series have radioisotopes that produce gamma rays with enough energy and intensity to be detected by gamma ray spectrometry, despite the fact that many naturally occurring elements have radioactive isotopes. This is because they are relatively abundant in the natural environment. Average crustal abundances of these elements quoted in the literature are in the range 2–2.5% K, 2–3 ppm U and 8–12 ppm Th. Radiometric surveys for geological mapping and mineral exploration use a variety of different methods and instruments. In the field, a spectrometer connected to a scintillation detector picks up gamma rays and their appropriate energies. Depending on the goal of the survey and the geological or environmental issue under investigation, a portable gamma ray spectrometer’s field method will be chosen. A portable gamma ray spectrometer’s response is influenced by the size, position, and shape of radioactive sources. The source-detector geometry must remain constant across all observations in order to produce accurate measurements along a traverse.

The detector needs to be kept at a low but constant height, or it should be positioned directly on the earth’s surface. This minimizes the effects of local variation in relief and radioactive element distribution. For a detector placed on the ground, the effective rock sample has a thickness of approximately 25 cm, a radius of 1 m, and a mass exceeding 100 kg. If the height of the detector is raised, the effective source increases rapidly in diameter from several meters to tens of meters depending on the energy of gamma rays [12,13]. Survey grids and traverse of spacings should reflect the expected strength, size and distribution of sources. For example, it is unlikely that small point sources will be detected on traverse tens of meters apart. On the other hand, regional traverses can give good estimates of the radioactivity of broad-scale lithological units.

### 2.4. Seismic method

The seismic method utilizes the propagation of waves through the earth and is the most commonly conducted geophysical survey for engineering and groundwater (some extent) investigation. According to [14] the seismic method is by far the most important geophysical technique in terms of cost effectiveness, high accuracy, high resolution and great penetration of which the method is capable. Reflection and refraction are the most commonly used seismic techniques. But in this field work studies seismic refraction method has been used as the primary to provide detailed information about subsurface layering and rock properties with their associated velocity and depth determination using seismic waves.

Seismic waves are in the form of packets of elastic strain energy that travel from a naturally or artificially generated source. It has two major components, the Body and Surface waves [15].

Surface waves are in the form of Rayleigh and these waves travel along the surface of the Earth with a more complicated particle motion and are responsible for damages during the release of energy earthquakes [16]. The seismic wave velocity varies from layer to layer depending on the density and elasticity of the subsurface material. The velocities of the seismic wave (Vp and Vs) in a homogeneous isotropic medium are given by:


$$V_{p}=\sqrt{\frac{K+\frac{\text{4}}{\text{3}\mu }}{\rho }}\text{ and }V_{s}=\sqrt{\frac{\mu }{\rho }}$$

Where ${\;V}_{p}$ p – wave, $V_{s}$ – secondary wave, K is the bulk modulus, μ is the shear modulus, ρ is the density of the material through which the wave propagates,

These speeds are controlled by a set of physical constants, called elastic parameters that describe the material [17], so that because of the dependence of seismic velocities on the elasticity and density of the material of the subsurface layer through which it is passing seismic refraction surveys also give a measure of material strengths and consequently it acts as an aid in assessing rock strength and rock quality [18].

The seismic energy generated by a seismic source (‘shot’) located on the surface radiates outward from the shot point spreading in all direction [18], it may either travel directly through upper layer (direct arrivals), or it may travel down to and then laterally along the high velocity layers (refracted arrivals) before bouncing up and coming back to the surface. According to [17] a suitable image (model) of physical properties is constructed based on a set of measured data through a mathematical framework providing by the inverse theory. If we represent some property of the subsurface (example velocity) by a set of model parameters m, then a set of data (example travel time) d can be predicted for a given source receiver array by line integration through the model. The relationship between data and model parameters forms the basis of any tomographic model;


d=Gm

**Fig 1:**
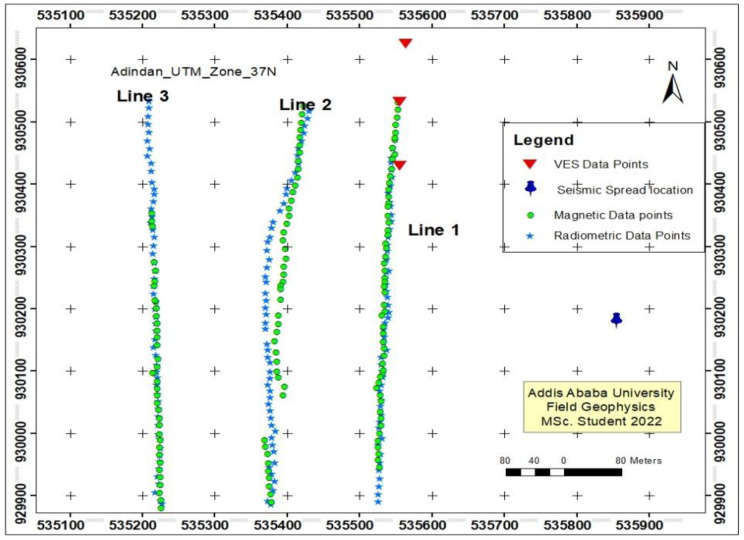
Layout of the Survey Line Awash Melkasa Agricultural Research Center.

*Where* d-vector of the observation G- Kernel matrix that relates the model to the observation m- Model parameter. The instruments used for collecting the seismic refraction data are Seistronix RAS-24 seismograph, battery source, Laptop computer to display the wave form, geophones to convert mechanical energy to electrical wave form, triggering cable and a 10 Kg sledge hammer and a metal plate as an input source. In this field work 24 channel seismic surveys were carried out with a geophone spacing of 3m. These geophones are connected to the seismograph via cable system unit that is controlled by a laptop computer. Moreover, a 10 kg sledge hammer and plate are connected to the trigger point, whose end goes to the seismograph. When taking measurements, at one shot point is done the same process continues at the next trigger position. The source was activated at five different points for the spread. The locations of the shots from the first geophone distance were -18m, -3m, 34.5m, 72m, and 87m for the case of Far Offset- forward, Near Offset- forward, Central shot (mid-line, split-spread shot point), near offset- reverse and Far Offset- reverse shots respectively (Fig 1).

## 3. Discussion and interpretation

### 3.1. Interpretation of VES data

The results of resistivity sounding survey are presented in the form of interpreted VES curves, pseudo depth map, geo-electric section and sliced stacked section for the purpose of qualitative assessments and vertical geo-electric section, permitting quantitative interpretations.

#### 3.1.1. Interpreted VES curves.

**Fig 2:**
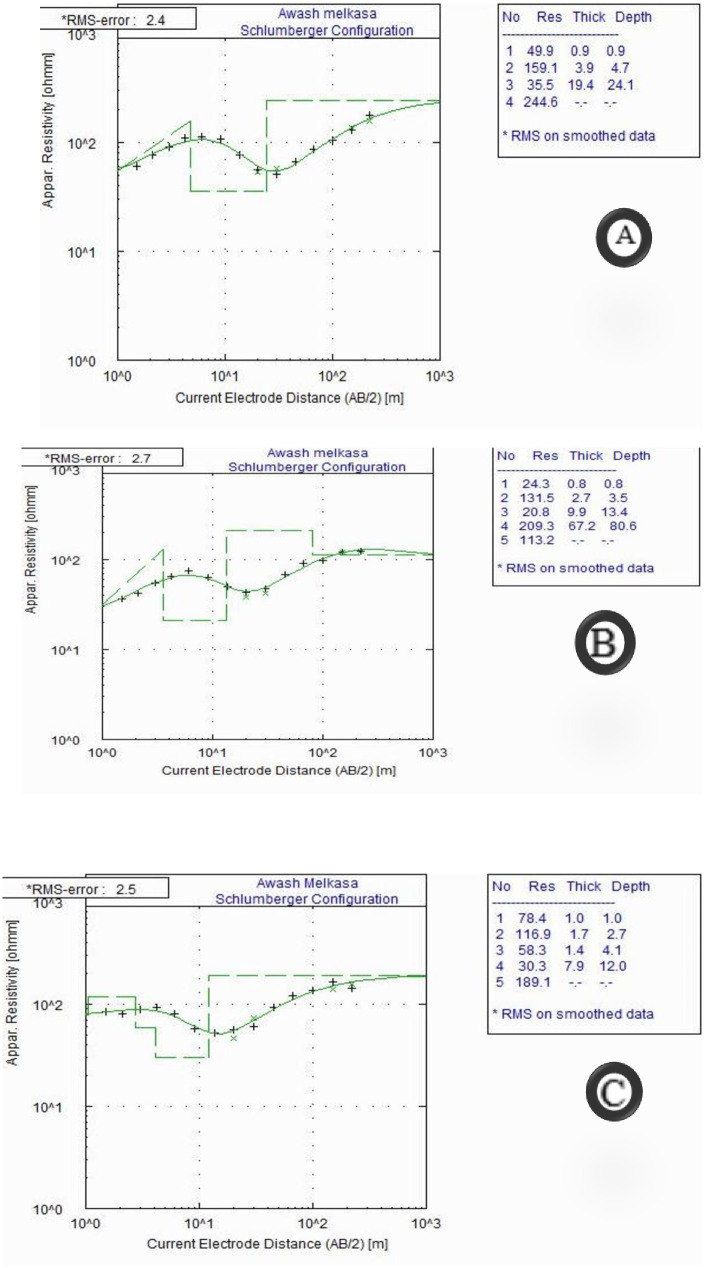
Interpreted sounding curves for a) VES 1 b) VES 2 and c) VES 3, Awash Melkasa Agricultural Research Center.

A very strong connection between the field data and the interpreted model sections is found for all three VES points from the interpreted field curves. An RMS error of 2.4 to 2.7% found in the sounding datasets to this. The interpreted VES curves from the survey traverses are used as an example, and they are shown in figure shown below. A 4–5 layer of the subsurface is shown to accurately represent the subsurface in the three sounding curves (using the AB/2 = 220m utilized for the survey) (Fig 2).

From the resistivity curves shown below, the smooth curve shows four different resistivity layers with different layer thicknesses. A very thin layer with low resistivity was at the top which indicates the top soil, followed by a thin layer with moderate resistivity indicating highly consolidated tuff, a thick layer with high conductivity indicting fractures ignimbrite and probable aquifer zone, and finally a very thick layer with low resistivity indicating highly fractured ignimbrite was observed from the processed data.

#### 3.1.2. Pseudo-depth resistivity section.

**Fig 3:**
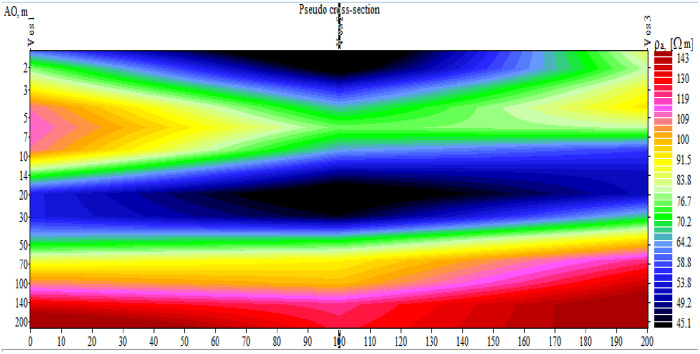
The Apparent Resistivity Pseudodepth Section of the VES Traverse line, Awash Melkasa Agricultural Research Center.

The distance between the VES points served as the x-axis for the pseudo-depth section, which was created using the AB/2 as the depth values to show the difference in resistivity values both horizontally and vertically (Fig 3). To determine the distribution of various resistivity values in the lateral and vertical directions, the pseudo-sections were built using the VES data along all survey lines. For the purpose of creating geo-electric sections, the qualitative interpretation of pseudo-sections provides a preliminary framework for the identification of various resistive materials and an analysis of relative resistivity variation. The pseudo–depth section reveals the distribution of subsurface resistivity and as indicated on the (Fig 3) the section shows resistivity variation of subsurface in five layers beneath each VES point.

#### 3.1.3. Geo-electric profile section.

**Fig 4:**
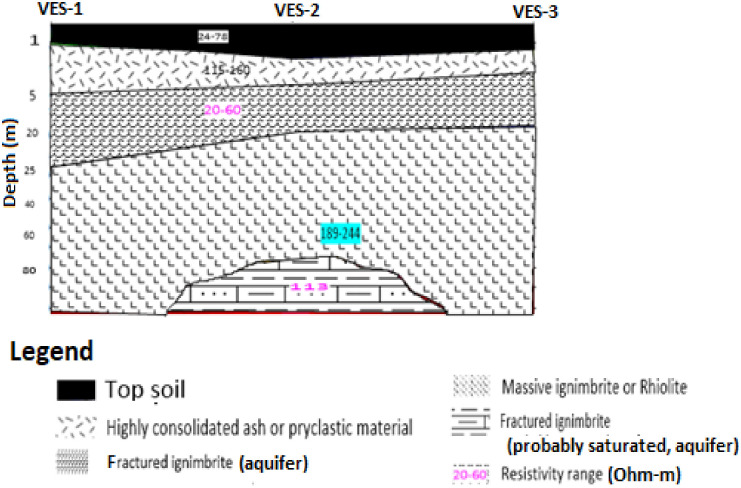
Geoelectric profile section along the VES traverse line, Awash Melkasa Agricultural Research Center.

The geoelectric section was prepared by the interpolation of layer thickness and resistivity variation of each layer using SURFER-16 software. The resulting geo-electric section constructed from the interpreted layer parameters of the all VES lying on this traverse is given in (Fig 4) below. The final result from one dimensional inversion of VES data along survey lines was used to construct the geo-electric sections in order to identify the distribution of different lithological units in the vertical direction. The difference in the resistivity values is due to the variation in the amount of grain size, type and degree of weathering and fracturing of the material beneath the measurement point.

The top geo-electric layer is the combination of three interpreted VES curve for simplification of the model. As indicated on the (Fig 4) the near-surface geo-electric layer has a resistivity range varying from 24–78 Ohm-m and this layer is interpreted as Top soil. The second layer has resistivity range of 115–159 Ohm-m and thickness variation of 1.7-3.96m this layer is interpreted as welded tuff, slightly weathered consolidated ash and Pyroclastic material. The third layer has a resistivity range of 20–60 Ohm-m and thickness variation of 9.3 to 19.4m. This layer is interpreted as moderately weathered and fractured Ignimbrite or slightly weathered and highly fractured and weathered ignimbrite. Due to its thick layer and low to intermediate resistivity range the layer is interpreted as saturated zone and probable aquifer zone.

The fourth layer has a resistivity value varying from 189 to 244 Ohm-m and this layer has infinite thickness beneath VES 1 and VES 3 and 80m beneath the second VES. The type of lithology of this layer may be un-fractured Ignimbrite or massive ignimbrite and also it may be Rhyolite due its high resistivity range. The last layer which is found only beneath the VES 2 is interpreted as low resistivity zone with resistivity value of about 113 Ohm-m and this layer is also interpreted as highly fractured and weathered Ignimbrite or this layer has good aquifers as compared to the above layer.

### 3.2. Interpretation of magnetic data

The Interpretation of magnetic data in equatorial areas is an ambiguous as smaller field intensity and horizontally directed ambient inducing field produces several complications [19]. According to [20], total magnetic anomalies are highly variable in shape and amplitude; they are almost always asymmetrical, sometimes appear complex even from simple sources, and usually show the combined effects of several sources. An infinite number of possible sources can produce a given anomaly, giving rise to the term ambiguity.

However, an ambiguity in interpretation of magnetic data can be precluded [19] using appropriate data enhancement techniques: All the magnetic data processing of the present study has been done using Oasis Montaj software (v 6.4.2).

#### 3.2.1. Total magnetic field intensity map.

**Fig 5:**
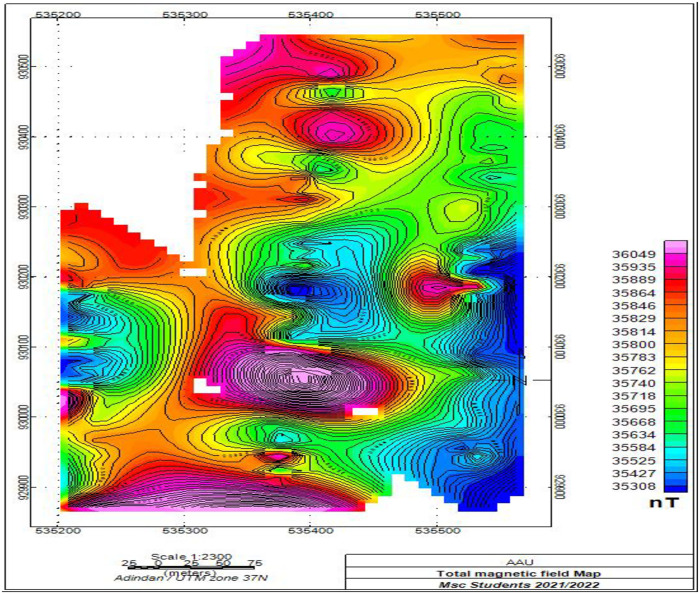
Total magnetic field intensity map of the study area, Awash Melkasa Agricultural Research Center.

The total magnetic field intensity map (Fig 5) of the study area is compiled by plotting all the magnetic data that are corrected for diurnal variation at their respective locations. The map is thought to show the variation in magnitude of the combined effect of rock magnetization and the dipole field over the survey area.

The total field intensity values over the survey area vary from a minimum of 35308nT to a maximum of 36049nT. The areal distribution of the major positive anomalies and the maximum difference (741nT) between the maximum and minimum intensity values revealed by the map indicate the presence of prominent anomalous geologic features in the survey area. Generally, the total magnetic field intensity map can be classified into three anomalous area of different intensity values. The first anomalous area is characterized by high total magnetic intensity values ranging from 36049nT to 35829 nT and associated with the locations of volcanic rocks specially scoria basalt. The second anomalous area are characterized by intermediate magnetic intensity values ranging from 35584 nT to 35814 nT and associated with pyroclastic deposits. The third anomalous localities are characterized by magnetic intensity values of less than 35525nT and associated with the Evidences indicate that these localities are occupied by thick accumulation of sediments.

#### 3.2.2. Total magnetic field anomaly map.

The magnetic anomaly map produced is the difference between the diurnally corrected total magnetic field and the determined value of the IGRF. The magnetic field recorded in the field using the Scintrex IGS-2 proton precession magnetometer is due to the effect of the Earth’s main magnetic field, the external field arising from solar activities and the anomalous magnetic field that arises from variations in subsurface geology. Magnetic data collected from low latitude is difficult to interpret due to the small magnetic field intensity and horizontal ambient field direction. At low magnetic latitudes, anomalies over magnetically susceptible bodies show negative values instead of positive and the anomalies largely depend on azimuthal direction [19] and therefore....

**Fig 6:**
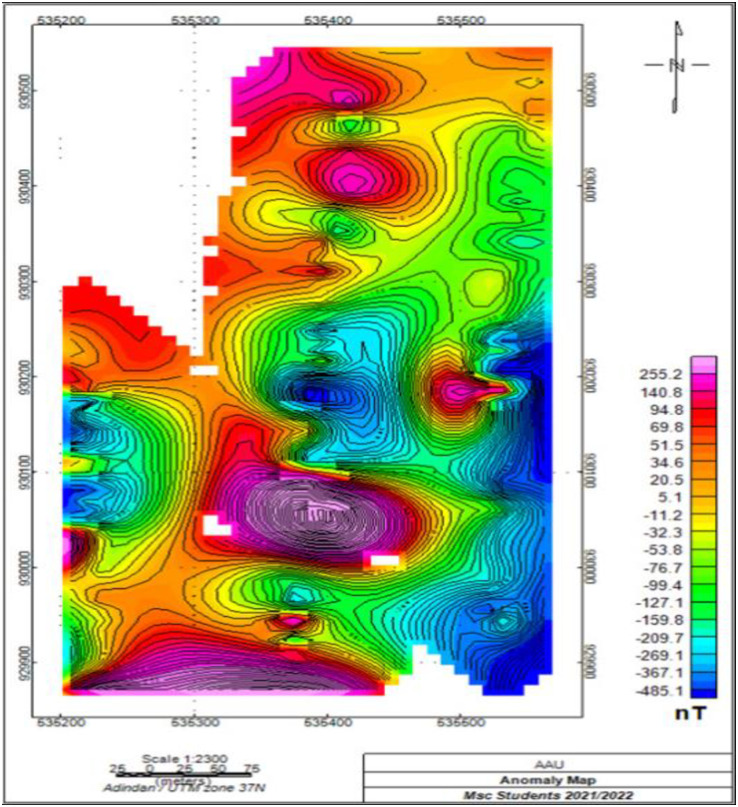
Total magnetic field anomaly map of the tudy area, Awash Melkasa Agricultural Research Center.

As shown in (Fig 6) of the study area indicate high total magnetic anomaly over the northern and southern part of the map which is caused by high magnetic susceptibility of rocks (basalt) which are expected to show high remanent magnetization as compared to other felsic volcanic rocks, reveals high total magnetic anomaly and the central, eastern, southeastern and western parts are characterized by low magnetic anomalies, which may be attributed to the low magnetic susceptibility of rocks or thick accumulation of sediments..

#### 3.2.3. Regional magnetic anomaly map.

The regional magnetic anomaly map was generated using a low pass filtering technique in Oasis Geosoft Montaj (v 6.4.2). This method effectively isolates regional trends by removing high-frequency noise.

**Fig 7:**
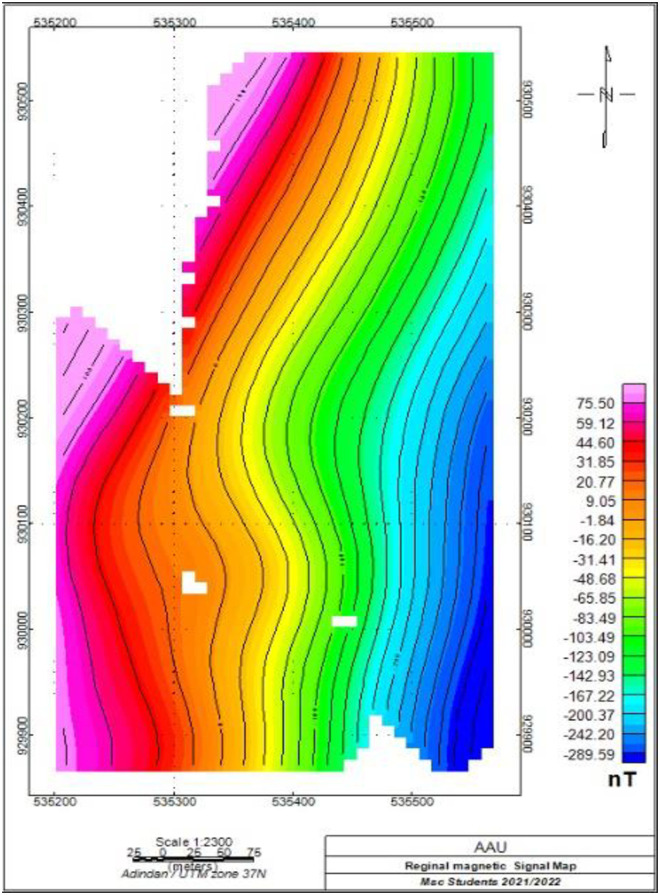
Regional magnetic anomaly map, Awash Melkasa Agricultural Research Center.

In this analysis, a specific frequency band was applied to differentiate regional anomalies from localized sources. The low pass filter emphasizes broader geological structures, revealing high susceptibility volcanic rocks in the western, southwestern, and northwestern areas. Intermediate anomalies appear in the central and south-central regions, while very low susceptibility is noted in the eastern part of the map in (Fig 7). The selected frequency band is crucial for accurately interpreting the subsurface geology.

#### 3.2.4. Analytical signal magnetic anomaly map.

According to [21] the analytical signal map of a potential field is produced by combining the 3-directional gradients of the potential field at location in use and given by;

$As(x,\;y,z)=\;\sqrt{{\left(\frac{\partial f}{\partial x}\right)}^{\text{2}}+{\left(\frac{\partial f}{\partial y}\right)}^{\text{2}}+{\left(\frac{\partial f}{\partial z}\right)}^{\text{2}}}$ Where, f is the magnetic field (residual magnetic) considered in the computation.

Generally, the interpretation of magnetic data is difficult at low magnetic latitudes due to the complex nature of magnetic field at the equator/low latitude. However, the magnitude of analytical signal map reveals maximum value over magnetic contacts regardless of the direction of magnetization and is always positive.

**Fig 8:**
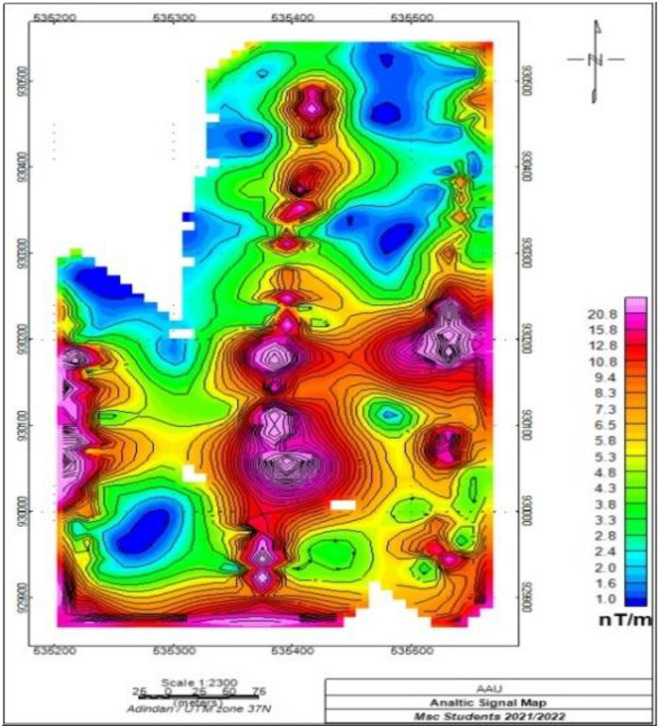
Magnetic analytic signal map of the study area.

The analytical signal magnetic map (Fig 8), obtained from the residual map, shows maxima at the edge of the source body, i.e., it shows structural/lithological discontinuity regardless of the direction of magnetization. It is easier to infer source position from analytical signal map as it enhances short wave length anomalies. The analytical signal map is highly related to the geology of the area and low peaks within the map are attributed to the response of lacustrine sediments. Susceptibility contrasts in the basaltic units in contact result in large analytic signal gradients as compared with the surrounding sediments and low magnetic susceptibility rock units like ignimbrite, tuffs, rhyolite and other pyroclastic volcanic rocks.

#### 3.2.5. Magnetic Tilt derivative map.

The tilt derivative map of the study area was created by applying a tilt derivative filter to the magnetic analytical signal map using Geosoft Oasis Montaj software. The computation process begins with data preparation, where the magnetic analytical signal map is used as the primary input. This map highlights variations in magnetic intensity across the study area.

**Fig 9:**
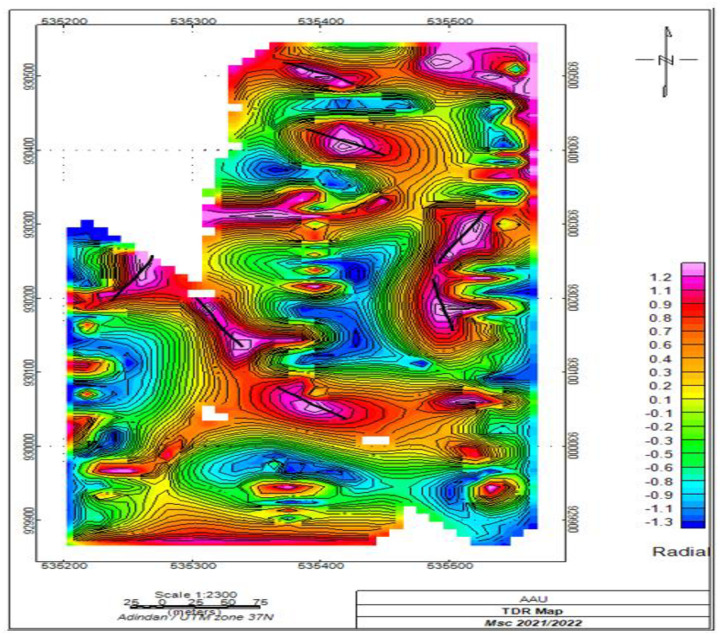
Tilt derivative magnetic map of the area.

Next, the tilt derivative filter is applied to the analytical signal map. This filter is crucial as it enhances the visibility of geological features by emphasizing edges and contacts, making it easier to identify structural boundaries. The resulting tilt derivative map displays distinct values: positive values over magnetic sources, a transition through zero at fault or contact locations, and negative values outside source zones. This pattern aids in pinpointing structural contacts and geological boundaries within the area. Upon analysis, the map reveals a network of faults oriented in N-S, NE-SW, and NW-SE directions shows in Fig 9. These orientations are correlated with the region’s tectonic history, particularly the presence of the Wonji fault belt. Overall, the tilt derivative map provides a clearer representation of structural features compared to the analytical signal map, facilitating a better understanding of the subsurface geology.

#### 3.2.6. Magnetic profile along Dera to Sire.

**Fig 10:**
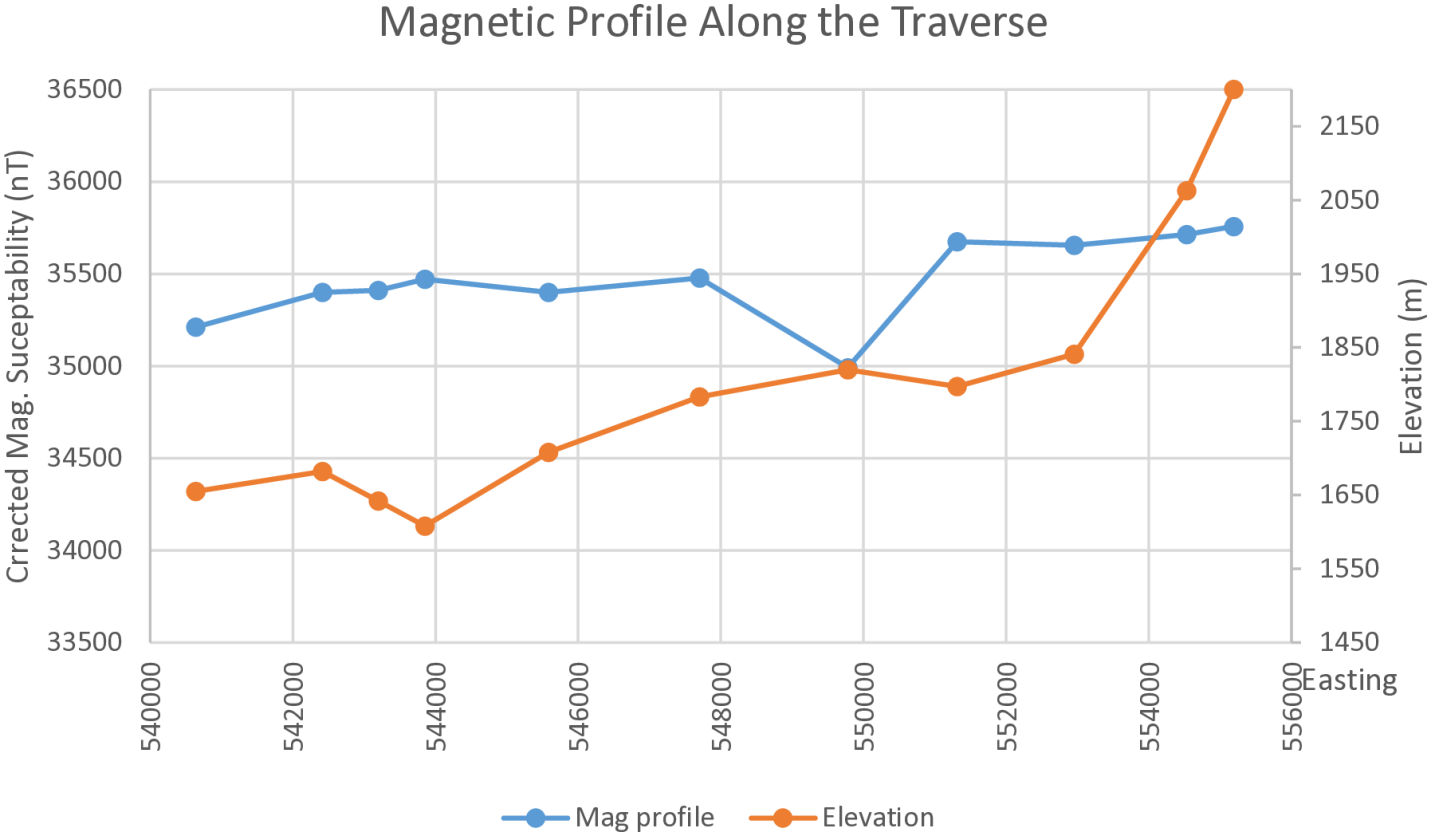
Magnetic profile line along Dera to Sire.

The observed total magnetic field intensity profile was created after the diurnal corrections. As shown in the profile given below (Fig 10), the total magnetic field intensity varies from 34991nT at fault plane (tuff/ignimbrite layer) on the first graben of the fault at the quarry site to 35757nT at the horst of Sire town. The total magnetic field intensity profile shows low anomalies at the graben of the fault plane as indicated by on the profile and higher at the horst of the fault. High total magnetic field intensity observed out of two major faults and where elevation is low to intermediate (graben).

### 3.3. Interpretation of radioactive data

**Fig 11:**
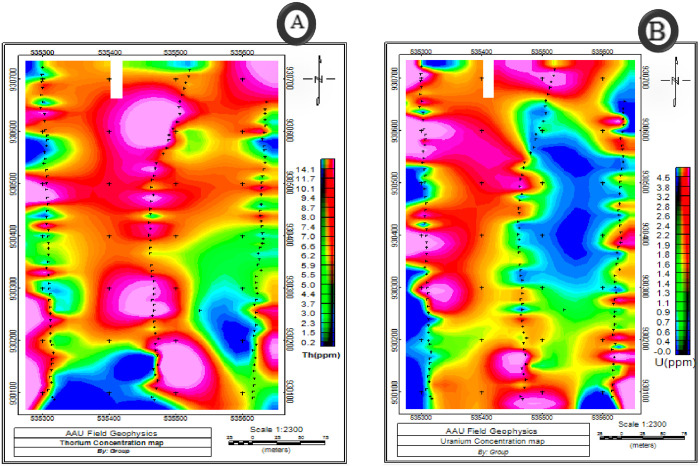
Equivalent radioactive element concentration map of A) Thorium B) Uranium.

**Fig 12:**
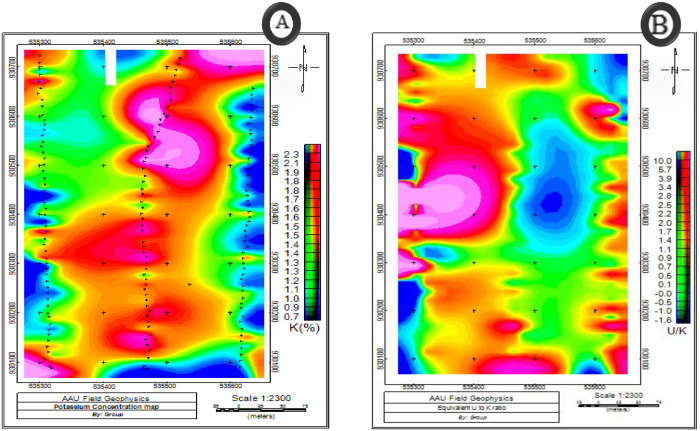
Equivalent concentration maps showing A) Potassium B) Uranium to potassium ratio.

The interpretation of gamma ray surveys should focus on enhancing and processing the data by applying knowledge of the mechanisms that control the distribution of radioactive elements in rocks and soils. Identifying any geological, topographical, climatic, and environmental aspects that might be pertinent to the data analysis is also a good idea. Three measured variables of radioactive element such as equivalent thorium (eTh, ppm) (Fig 11A), equivalent uranium (eU, ppm) (Fig 11B) and potassium (K, %) (Fig 12A) maps are prepared from the collected field data.

High intensity zones have been shown on equivalent maps of Thorium and Uranium as beginning in the area’s northern to northwestern region. Some areas in the central to eastern half and to the southwestern parts had low Thorium and uranium concentrations, whereas soil-covered areas in the southernmost part had high quantities. Acid and intermediate extrusive igneous rocks are found in areas with high Thorium and uranium concentration. Additionally, low Thorium and uranium levels point to the presence of felsic rocks that have weathered.

High Potassium content in the central section of the study area suggests the presence of potassium rich units and is defined by the felsic extrusive igneous rocks. Because of the development of gravel roads basic extrusive rocks like scoria and basalts that are carried from other locations are likely present on the eastern, western, and southern boundaries of the study area and this is indicated by the presence of Low potassium content on the potassium concentration map of the study area (Fig 12A).

**Fig 13:**
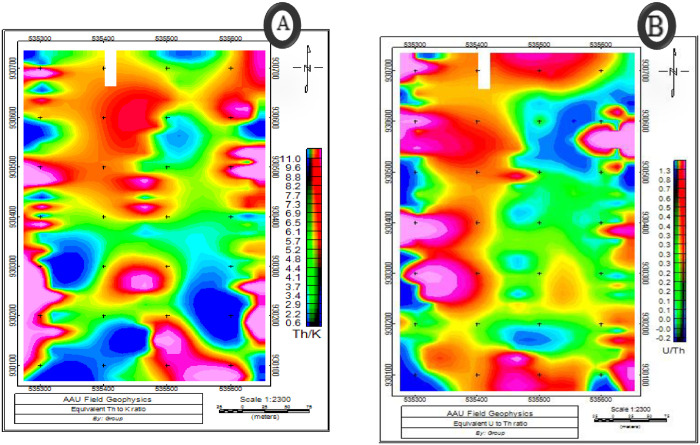
Maps showing A) Thorium to Potassium ratio B) Uranium to Thorium ratio.

According to [21], ratio patterns can magnify small variations in elemental concentrations resulting from lithological changes or alteration processes related to mineralization. The U/ Th ratio (Fig 13B), for example, decreases in weathered rock since uranium is easily oxidized to a water-soluble form and Thorium has no soluble ion and therefore tends to remain with the parent rock or is transported over relatively short distances in the form of solid mineral grains [22].

A low Th/k ratio is good indicator of potassic alterations and it is indicated on the (Fig 13A). Which shows reliable predictor of potassium alteration in the rocks. Thus, the profiles that include this ratio are often very useful for picking specific target anomalies for ground follow-up in areas of exploration. As indicated on the (Fig 13A) the western, south western and south-eastern parts of the study area shows low Th/k values which indicate zones of potassic alteration. Fig 13B also shows the ratio between the U and Th concentration in the area.

### 3.4. Interpretation of seismic refraction data

The seismic refraction data were processed using Seism-Imager/2D software. The first step involved utilizing the Pickwin95 module to determine the first arrival times of seismic waves. This module accurately identifies the initial wave arrivals, which are crucial for subsequent analysis. Once the first arrival times were established, the data were imported into the Plotrefa module of Seism-Imager/2D. This module employs tomography modeling algorithms to create a p-wave velocity model based on the collected data.

The resulting velocity model was then interpreted in the context of the geological characteristics of the study area. By correlating the model parameters with known geological features, insights into the subsurface structure and composition were obtained. Overall, this systematic approach allows for an accurate determination of layer velocities, facilitating a better understanding of the geological framework.

#### 3.4.1. Velocity layer model of the spread.

**Fig 14:**
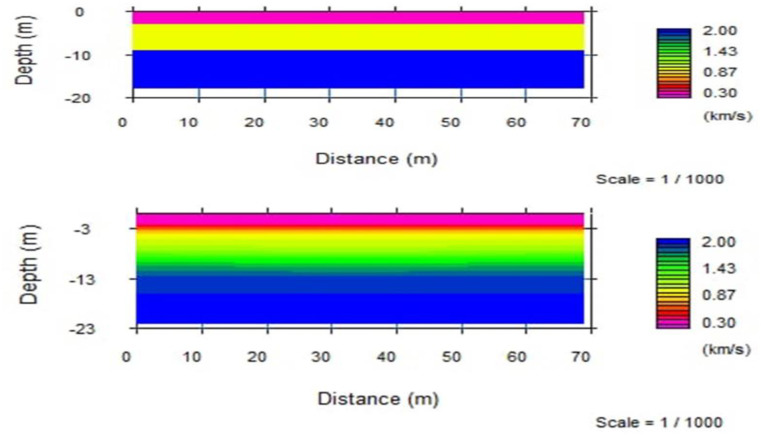
Time-term and 2D seismic tomography velocity models for spread.

**Fig 15:**
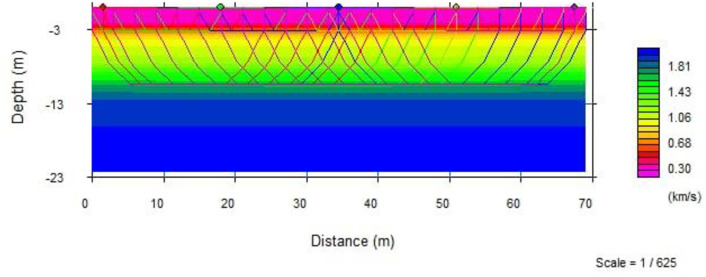
2D seismic tomography velocity models for spread with ray path.

This spread is 69m long and runs in E-W direction (Fig 14). Travel-time inversion method was used to generate the 2D subsurface model. The velocity model represents seismic velocities between 0.3 km/s and 2 km/s. The top most layer shows low P-wave velocity that vary between 0.3–0.7 km/s and is about 3m thick with no difference in thickness along the spread. The low velocity indicates that the top layer is essentially composed of soil deposits. The p-wave velocity of the second layer is varying from 0.75 km/s – 1.2 km/s with depth about 3-9m and it’s probably interpreted as unconsolidated pyroclastic ignimbrite. The p-wave velocity in the third layer associated with 9 - 18m in depth is relatively high 2 km/s and this layer is probably moderately weathered and fractured ignimbrite (Fig 15).

## 4. Conclusion

The interpretation of combined geophysical methods such as electrical, magnetic, radiometric, and seismic alongside geological data has provided valuable insights into the geodynamic setting, structures, and anomalous bodies in the Awash Melkesa area. The vertical electrical soundings (VES) revealed a low resistivity zone interpreted as a highly fractured and weathered ignimbrite, indicating potential as an aquifer. However, the study has certain limitations. The reliance on geophysical methods can sometimes lead to ambiguous interpretations, particularly in complex geological settings where multiple factors influence the measurements. Additionally, the spatial resolution of geophysical surveys may not capture all subsurface features, potentially overlooking smaller or more intricate structures.

Despite these limitations, the approach adopted in this study has several positive aspects. The integration of multiple geophysical techniques enhances the reliability of the interpretations, allowing for a comprehensive understanding of the subsurface geology. The identification of NE-SW trending structures associated with the Wonji fault belt and the analysis of radioactive concentrations in different rock types highlight the effectiveness of the combined methodologies. Overall, this multi-faceted approach not only improves the accuracy of geological interpretations but also aids in resource exploration and development, setting a foundation for future studies in similar geological contexts.

## Supporting information

S1 File Geophysical data. (RAR)
